# Light-Intensity Physical Activity and Cardiometabolic Biomarkers in US Adolescents

**DOI:** 10.1371/journal.pone.0071417

**Published:** 2013-08-09

**Authors:** Valerie Carson, Nicola D. Ridgers, Bethany J. Howard, Elisabeth A. H. Winkler, Genevieve N. Healy, Neville Owen, David W. Dunstan, Jo Salmon

**Affiliations:** 1 Faculty of Physical Education and Recreation, University of Alberta, Edmonton, Alberta, Canada; 2 Centre for Physical Activity and Nutrition Research, School of Exercise and Nutrition Sciences, Deakin University, Melbourne, Victoria, Australia; 3 Baker IDI Heart and Diabetes Institute, Melbourne, Victoria, Australia; 4 Cancer Prevention Research Centre, School of Population Health, The University of Queensland, Brisbane, Queensland, Australia; 5 Melbourne School of Population and Global Health, The University of Melbourne, Melbourne, Victoria, Australia; 6 Department of Medicine, Alfred Hospital, Monash University, Melbourne, Victoria, Australia; 7 School of Sport Science, Exercise and Health, The University of Western Australia, Perth, Western Australia, Australia; 8 Department of Epidemiology and Preventive Medicine, Monash University, Melbourne, Victoria, Australia; Innsbruck Medical University, Austria

## Abstract

**Background:**

The minimal physical activity intensity that would confer health benefits among adolescents is unknown. The purpose of this study was to examine the associations of accelerometer-derived light-intensity (split into low and high) physical activity, and moderate- to vigorous-intensity physical activity with cardiometabolic biomarkers in a large population-based sample.

**Methods:**

The study is based on 1,731 adolescents, aged 12–19 years from the 2003/04 and 2005/06 National Health and Nutrition Examination Survey. Low light-intensity activity (100–799 counts/min), high light-intensity activity (800 counts/min to <4 METs) and moderate- to vigorous-intensity activity (≥4 METs, Freedson age-specific equation) were accelerometer-derived. Cardiometabolic biomarkers, including waist circumference, systolic blood pressure, diastolic blood pressure, HDL-cholesterol, and C-reactive protein were measured. Triglycerides, LDL- cholesterol, insulin, glucose, and homeostatic model assessments of β-cell function (HOMA-%B) and insulin sensitivity (HOMA-%S) were also measured in a fasting sub-sample (n = 807).

**Results:**

Adjusted for confounders, each additional hour/day of low light-intensity activity was associated with 0.59 (95% CI: 1.18–0.01) mmHG lower diastolic blood pressure. Each additional hour/day of high light-intensity activity was associated with 1.67 (2.94–0.39) mmHG lower diastolic blood pressure and 0.04 (0.001–0.07) mmol/L higher HDL-cholesterol. Each additional hour/day of moderate- to vigorous-intensity activity was associated with 3.54 (5.73–1.35) mmHG lower systolic blood pressure, 5.49 (1.11–9.77)% lower waist circumference, 25.87 (6.08–49.34)% lower insulin, and 16.18 (4.92–28.53)% higher HOMA-%S.

**Conclusions:**

Time spent in low light-intensity physical activity and high light-intensity physical activity had some favorable associations with biomarkers. Consistent with current physical activity recommendations for adolescents, moderate- to vigorous-intensity activity had favorable associations with many cardiometabolic biomarkers. While increasing MVPA should still be a public health priority, further studies are needed to identify dose-response relationships for light-intensity activity thresholds to inform future recommendations and interventions for adolescents.

## Introduction

In order to improve health, the World Health Organization recommends that adolescents should accumulate at least 60 minutes of daily physical activity that is of moderate-to vigorous-intensity (MVPA) [1]. This includes activities such as brisk walking, bicycling, and soccer [2]. Many countries and organizations have made similar recommendations [3]–[5]. However, recent findings using objective measurement in large population samples show that a high proportion of adolescents are not participating in sufficient MVPA on a daily basis [6], [7]. For instance, 92% of adolescents assessed by accelerometer in the US National Health and Nutrition Examination Survey (NHANES) were classified as not meeting current physical activity guidelines [6]. Similar statistics were reported for Canadian adolescents in the Canadian Health Measures Survey [7].

For adolescents, physical activities at the higher end of the intensity continuum have stronger health benefits, relative to physical activities of lower intensities [8]–[12]. However, the majority of the evidence that has informed current physical activity guidelines has been generated from studies that have focused on the health associations with physical activities of at least moderate intensity [8], [13]. Thus, the minimal physical activity intensity that would confer health benefits among adolescents is unknown. This is of considerable public health interest because reducing sedentary pursuits by increasing time spent in light-intensity activities may be a feasible and potentially beneficial first step for the large number of adolescents not meeting physical activity guidelines, especially those participating in no or minimal physical activity. Furthermore, MVPA only accounts for a small proportion of waking hours among adolescents [6], [7], even if they are accumulating the recommended 60 minutes throughout the day [14]. In contrast, there may be substantially more opportunities to increase light-intensity activities in a given day through incidental activities such as light ambulatory movement [14].

Capturing activity across the intensity spectrum, inclusive of light-intensity, is one of several advantages of measuring physical activity objectively with accelerometers, relative to the use of self- or proxy-report measures [15], [16]. Light-intensity activities range from static (e.g., standing) to dynamic (e.g., slow walking) [2], [17]. Of the limited number of studies that have investigated the relationships of accelerometer-derived light-intensity activity with health outcomes among adolescents [7], [12], [18]–[22], most have not observed beneficial associations with weight status [7], [12], [19]–[21] nor with other cardiometabolic biomarkers [12], [22]. One potential reason for the null findings is that the full range of light-intensity activities (i.e., static to dynamic) have been categorized into one light-intensity variable in previous studies. Given that health benefits appear to increase with activity intensity, it is possible that the more dynamic activities on the higher end of the light-intensity continuum may have stronger associations with cardiometabolic biomarkers, compared with the more static activities on the lower end of the light-intensity continuum. Consequently, to further understand the relationships of lower intensities of activity with adolescents’ health, it is important to distinguish between the lower and upper components of the light-intensity activity spectrum.

The purpose of the study was to examine the associations of accelerometer-derived low light-intensity physical activity (LLPA), high light-intensity physical activity (HLPA) and MVPA with cardiometabolic biomarkers in a large population-based sample of US adolescents.

## Methods

### Participants

NHANES is a large ongoing national surveillance study conducted in the United States [23]. NHANES uses a complex multi-stage probability sampling procedure to select a representative sample of the US population from all age groups. Data are collected through home interviews and physical exams, which are conducted in mobile examination centers [23]. Accelerometer data are available for the 2003/04 and 2005/06 NHANES cycles. Out of the 4,591 NHANES participants aged 12–19, a total of 3,779 wore an accelerometer and were eligible for inclusion in this study.

### Ethics Statement

Ethics approval was obtained from the National Centre for Health Statistics Research Ethics Review Board. Written informed consent was obtained from all participants and their parents/guardians if <18 years old.

### Physical Activity

Physical activity variables were derived from the uniaxial ActiGraph model 7164 accelerometer (ActiGraph, Ft. Walton Beach, FL). Participants were asked to wear the accelerometer on their right hip for seven consecutive days except when sleeping or during water-based activities. Data were collected in one minute epochs with NHANES survey collaborators excluding unreasonable or biologically implausible values [23]. Non-wear time was defined as a period of ≥60 minutes of continuous zero counts [24]. Only participants with ≥10 hours of wear time per day for a minimum of four days, including at least one weekend day, were included in the analyses [25]. Sedentary time was classified as <100 counts per minute (cpm) [16], [26], [27] and MVPA was classified as ≥4 metabolic equivalents (METs), according to Freedson’s age-specific regression equation [28]. Calibration studies indicate that a 4 MET threshold represents brisk walking in adolescents, which is a standard marker of moderate-intensity physical activity [16]. The remaining time was classified as either LLPA (100–799 counts/min) or HLPA (800 counts/min to <4 METs). The 800 cpm threshold was chosen because recent studies have indicated that this published sedentary cut-point captures both sedentary time and static light-intensity activities such as standing [26]. The Freedson regression equation was not used to isolate LLPA because prediction equations have been shown to be inaccurate for lower intensity activities [17]. To adjust for wear time, accelerometer-derived variables were standardized by using the residuals obtained when regressing the variables on wear time [29].

### Cardiometabolic Biomarkers

Waist circumference, systolic and diastolic blood pressure, HDL cholesterol, and C-reactive protein were examined in the full sample. Additionally, triglycerides, LDL cholesterol, plasma glucose, plasma insulin, and homeostatic model assessments of β–cell function (HOMA-%B) and insulin sensitivity (HOMA-%S) were examined in a sub-sample of participants who attended the morning examination and provided fasting blood measures. Furthermore, 2 hour plasma glucose was examined; however, this measure was only available in the 2005/2006 fasting sub-sample. Only participants who reported fasting for ≥8 hours were included in the fasting subsample analyses [23]. Detailed descriptions of these measurements are available through the NHANES website [23].

### Covariates

Age, sex, race (as classified by investigators: non-Hispanic White, non-Hispanic Black, Mexican American, other), socioeconomic status (SES), smoking (yes or no), total dietary intake, saturated fat, and sodium intake were considered as covariates. SES was measured using the poverty-to-income ratio, which is a ratio between family income and poverty threshold [23]. Smoking was assessed by asking participants if they had previously tried cigarette smoking. Total dietary intake, saturated fat and sodium intake were derived from a 24 hour recall. Saturated fat (≤10% or >10% of total calories) and sodium (≤2300 or >2300 mg/day) were dichotomized based on US dietary guidelines [30].

### Statistical Analysis

Analyses were conducted using SAS version 9.2 (SAS Institute Inc., Cary, NC) and accounted for the complex design and sample weights of NHANES. Since missing data (mostly due to accelerometer non-compliance) was substantial, and adolescents included in the full analyses were slightly younger (0.4 years; *P*<0.05) compared to adolescents that were excluded due to missing data, sample weights were re-weighted for non-response to achieve a representative sample. The assumption of normality in the regression models for biomarkers was assessed by examining residuals. Waist circumference, C-reactive protein, triglycerides, LDL-cholesterol, insulin, HOMA-%B, and HOMA-%S were log-transformed. Separate multiple linear regression models were used to examine the association between each physical activity exposure and cardiometabolic biomarkers. Results are expressed as the effect on the mean levels of each biomarker (or relative rates, for log-transformed outcomes) for each additional hour/day of physical activity. Model 1 adjusted for potential confounders identified from the literature (age, sex, ethnicity, SES, smoking, total energy intake, sodium, and saturated fat) [12], [22], [31]. Model 2 adjusted additionally for waist circumference, which may be a confounder but also may be a mediator.

Additional analyses further adjusted for MVPA in the LLPA and HLPA final models to determine if associations with cardiometabolic biomarkers were independent of MVPA. Sedentary behavior was not included in the models because it was highly correlated with both LLPA and HLPA (*r*>0.80). Further, a sensitivity analysis was conducted to determine whether any associations of HLPA and MVPA with cardiometabolic biomarkers were consistent when MVPA was defined as ≥3 METs. While there is established precedence in the pediatric literature to define MVPA as ≥4 METs [16], [32], ≥3 METs has been used in previous studies [16]. Finally, moderation analyses were conducted to examine whether associations differed by age, sex, race, and waist circumference. Statistical significance was set at *P*<0.05 for main effects and *P*<0.001 for interactions. Given our main research question, a strict criterion was used for interactions to minimize type 1 error.

## Results

Of the 3,779 eligible participants, 1,633 were excluded due to incomplete accelerometer data and an additional 415 were excluded due to incomplete information on outcome or covariate measures. In total, 1,731 were included in the full analyses, 807 in the fasting analyses, and 359 for the 2 hour plasma glucose analyses. With reweighting to correct for non-response, the full sample and fasting subsample both closely resembled the US adolescent population (Table 1). Weighted to the population, median age was 15 years, approximately half of the sample was male, 64% were non-Hispanic White, 15% were non-Hispanic Black, and 11% Mexican-American. The median time spent in each physical activity category diminished with intensity: 250, 102 and 19 minutes/day in LLPA, HLPA and MVPA, respectively.

**Table 1:** Participant Characteristics.

Variables	Total Sample (N = 4455)	Full sample (N = 1731)	Fasting sub-sample (N = 807)
**Socio-demographic**			
Age (years)	14.9 (13.0–16.9)	14.5 (12.8–16.6)	14.7 (12.8–16.8)
Sex (%)			
Male	50.9	50.9	50.9
Female	49.1	49.1	49.1
Race (%)			
Non-Hispanic White	61.7	63.8	62.8
Non-Hispanic Black	16.1	15.1	15.0
Mexican-American	12.7	11.3	11.3
Other	9.5	9.8	10.9
Poverty to income ratio	2.5 (1.1–4.1)	2.5 (1.2–4.1)	2.5 (1.2–4.2)
**Behavioral**			
Ever tried smoking (%)			
Yes	–	33.3	35.0
No	–	66.7	65.0
Total energy intake (kcal)	–	2175.0 (1599.5–2778.5)	2232.2 (1635.5–2873.5)
Sodium Intake (%)	–		
≤2300 mg/day	–	24.3	23.2
>2300 mg/day	–	75.7	76.8
Saturated Fat (%)	–		
≤10% of total calories	–	33.0	37.3
>10% of total calories	–	67.0	62.7
**Accelerometer-derived variables**			
Total wear time (min/day)	–	862.5 (801.9–928.1)	868.1 (803.0–929.7)
LLPA (min/day)†	–	249.9 (210.1–296.2)	244.0 (204.3–296.8)
HLPA (min/day)†	–	102.2 (75.0–130.1)	98.9 (71.1–127.8)
MVPA (min/day)†	–	18.9 (9.4–37.2)	18.0 (9.0–35.8)
**Biomarkers**			
Waist Circumference (cm)	–	76.9 (70.9–86.1)	–
Systolic Blood Pressure (mmHg)	–	108.8 (101.6–114.8)	–
Diastolic Blood Pressure (mmHg)	–	59.9 (53.0–66.9)	–
HDL Cholesterol (mmol/L)	–	1.3 (1.1–1.5)	–
C-reactive Protein (mg/dL)	–	0.04 (0.01–0.11)	–
Trigycerides (mmol/L)	–	–	0.8 (0.6–1.1)
LDL Cholesterol (mmol/L)	–	–	2.3 (1.8–2.7)
Plasma Glucose (mmol/L)	–	–	5.0 (4.8–5.3)
Insulin (pmol/L)	–	–	53.2 (34.2–76.5)
HOMA-%B (N = 768)	–	–	96.5 (74.6–128.6)
HOMA-%S (N = 768)	–	–	94.8 (68.0–138.7)

LLPA = low light-intensity physical activity; HLPA = high light-intensity physical activity; MVPA = moderate- to vigorous-intensity physical activity; HDL = High-density lipoprotein cholesterol; LDL = Low-density lipoprotein cholesterol; HOMA-%B = homeostatic model assessments of β–cell function; and HOMA-%S homeostatic model assessments of insulin sensitivity. Data presented as median (inter-quartile range) for continuous variables and % for categorical variables.

† Corrected for wear time using the residuals method, with cutpoints (in order) of 100 to 799 counts per minute (cpm), 800 cpm to 4METs (Freedson age-specific equation) and ≥4+ METs.

Associations of physical activity with cardiometabolic biomarkers in the full sample are shown in Table 2. There were beneficial associations of LLPA with diastolic blood pressure in the fully adjusted model (model 2). Each additional hour/day of LLPA was associated with 0.59 (95% CI: 1.18–0.01) mmHG lower diastolic blood pressure. There were also beneficial associations of HLPA with diastolic blood pressure and HDL-cholesterol. More specifically, in the fully adjusted model, each additional hour/day of HLPA was associated with 1.67 (95% CI: 2.94–0.39) mmHG lower diastolic blood pressure and 0.04 (0.001–0.07) mmol/L higher HDL-cholesterol. For MVPA, there were beneficial associations with waist circumference and systolic blood pressure in the fully adjusted models. Each additional hour/day of MVPA was associated with 3.54 (5.73–1.35) mmHG lower systolic blood pressure. After back-transforming waist circumference from the log scale, an additional hour/day of MVPA was associated with 5.49 (1.11–9.77) % lower waist circumference (equivalent at the mean to approximately 4 cm).

**Table 2:** Associations of time spent in LLPA, HLPA, and MVPA with cardiometabolic biomarkers in the full sample (n = 1731).

	LLPA (hour/day)	HLPA (hour/day)	MVPA(hour/day)
	β (95% CI)	β (95% CI)	β (95% CI)
Waist Circumference (cm)†			
Model 1	−0.001 (−0.012, 0.009)	0.008 (−0.008, 0.024)	−**0.052 (**−**0.093,** −**0.011)** *
Systolic blood pressure (mmHG)			
Model 1	0.373 (−0.636, 1.382)	−0.448 (−1.383, 0.489)	−**4.531 (**−**7.113,** −**1.950)** *
Model 2	0.401 (−0.649, 1.451)	−0.607 (−1.606, 0.392)	−**3.541 (**−**5.732,** −**1.350)** *
Diastolic Blood Pressure (mmHG)			
Model 1	**−0.596 (−1.188, −0.005)** *	**−1.640 (−2.959, −0.321)** *	−1.820 (−4.353, 0.714)
Model 2	−**0.592 (**−**1.176, −0.007)** *	**−1.667 (−2.942, −0.385)** *	−1.677 (−4.189, 0.835)
HDL-cholesterol (mmol/L)			
Model 1	0.009 (−0.013, 0.030)	0.031 (−0.008, 0.071)	0.085 (0.024, 0.195)
Model 2	0.008 (−0.013, 0.028)	**0.037 (0.001, 0.073)** *	0.050 (−0.049, 0.149)
C-reactive protein (md/dL) †			
Model 1	−0.012 (−0.089, 0.064)	0.081 (−0.061, 0.224)	−0.019 (−0.377, 0.339)
Model 2	−0.007 (−0.070, 0.056)	0.052 (−0.078, 0.181)	0.176 (−0.125, 0.477)

LLPA = low light-intensity physical activity; HLPA = high light-intensity physical activity; MVPA = moderate- to vigorous-intensity physical activity; and HDL = High-density lipoprotein cholesterol.

* ***P*<0.05;**

† Log transformed; β (95% CI) = unstandardized regression coefficients and 95% confidence intervals.

Model 1 is adjusted for age, sex, ethnicity, SES, smoking, total energy intake, sodium, and saturated fat; Model 2 is adjusted for confounders in model 2 and waist circumference.

Associations of physical activity with cardiometabolic biomarkers in the fasting sub-sample are shown in Table 3. No associations were observed between LLPA or HLPA with any assessed biomarkers. However, there were beneficial associations of MVPA with insulin, and HOMA-%S. In the fully adjusted model, after back-transforming insulin and HOMA-%S from the log scale, each additional hour/day of MVPA was associated with 25.87 (6.08–49.34) % lower insulin (equivalent at the mean to approximately 17 mmol/L), and a 16.18 (4.92–28.53) % higher HOMA-%S.

**Table 3:** Associations of time spent in LLPA, HLPA. and MVPA with cardiometabolic biomarkers in the fasting sub-sample (n = 807).

	LLPA (hour/day)	HLPA (hour/day)	MVPA (hour/day)
	β (95% CI)	B (95% CI)	B (95% CI)
Triglycerides (mmo1/L)†			
Model 1	0.004 (−0.012, 0.019)	0.009 (−0.010, 0.028)	0.018 (−0.058, 0.094)
Model 2	0.003 (−0.012, 0.019)	0.009 (−0.010, 0.029)	0.021 (−0.056, 0.098)
LDL**−**cholesterol (mmol/L)†			
Model 1	−0.007 (−0.032, 0.019)	0.005 (−0.010, 0.037)	0.017 (−0.111, 0.144)
Model 2	−0.007 (−0.031, 0.017)	0.005 (−0.024, 0.034)	0.025 (−0.202, 0.152)
Plasma Glucose (mmol/L)			
Model 1	0.004 (−0.005, 0.013)	0.013 (−0.001, 0.027)	−0.015 (−0.046, 0.016)
Model 2	0.004 (−0.005, 0.013)	0.013 (−0.001, 0.027)	−0.013 (−0.043, 0.017)
Insulin (pmol/L)†			
Model 1	−0.016 (−0.078, 0.046)	−0.024 (**−**0.127, 0.080)	**−0.300 (−0.545, −0.051)** *
Model 2	−0.018 (−0.060, 0.025)	−0.024 (−0.108, 0.061)	**−0.230 (−0.401, −0.059)** *
HOMA**−**%B (n = 768)†			
Model 1	0.001 (−0.044, 0.045)	−0.022 (−0.001, 0.048)	−0.111 (−0.256, 0.034)
Model 2	−0.002 (−0.033, 0.030)	−0.022 (−0.074, 0.031)	−0.077 (−0.179, 0.020)
HOMA**−**%S (n = 768)†			
Model 1	−0.013 (−0.075, 0.047)	−0.013 (−0.110, 0.084)	**0.203 (0.007, 0.401)** *
Model 2	−0.010 (−0.050, 0.029)	−0.013 (−0.09, 0.060)	**0.150 (0.048, 0.251)** *
**OGTT sub−sample (n = 359)**			
2h plasma glucose, mmol/l			
Model 1	−0.092 (−0.346, 0.163)	−0.109 (−0.452, 0.234)	−0.511 (−1.281, 0.258)
Model 2	−0.111 (−0.334, 0.113)	−0.114 (−0.417, 0.188)	−0.450 (−1.111, 0.212)

LLPA = low light-intensity physical activity; HLPA = high light-intensity physical activity; MVPA = moderate- to vigorous-intensity physical activity; LDL = Low-density lipoprotein cholesterol; HOMA-%B = homeostatic model assessments of β–cell function; HOMA-%S homeostatic model assessments of insulin sensitivity; and OGTT = Oral glucose tolerance test.

* ***P*<0.05;**

† Log-transformed; β (95% CI) = unstandardized beta coefficients and 95% confidence.

Model 1 is adjusted for age, sex, ethnicity, smoking, total energy intake, sodium, and saturated fat; Model 2 is adjusted for confounders in model 2 and waist circumference.

The associations of LLPA with diastolic blood pressure (β = –0.54 [95% CI: –1.09, 0.02]; *P* = 0.06) and HLPA with HDL-cholesterol (0.03 [–0.001, 0.07]; *P* = 0.06) became borderline non-significant after further adjusting for MVPA. Conversely, the beneficial associations of HLPA with diastolic blood pressure (−1.65 [−2.98, −0.33]) remained. In addition, associations of HLPA with diastolic blood pressure and HDL-cholesterol remained when MVPA was defined as ≥3 METs (See Table S1). With the exception of insulin and HOMA-%S, statistical conclusions regarding associations of MVPA with biomarkers were identical using the ≥3 and ≥4 MET thresholds (See Tables S1 and S2). No interactions by age, sex, race, or waist circumference were observed.

## Discussion

To better understand the relationship of lower intensities of physical activity with health, this study examined associations of accelerometer-derived LLPA and HLPA as well as MVPA with cardiometabolic biomarkers in a large, population-based sample of US adolescents. LLPA and HLPA had beneficial associations with diastolic blood pressure. HLPA also had beneficial associations with HDL-cholesterol. MVPA had beneficial associations with many of the biomarkers, including those related to adiposity (waist circumference), lipid metabolism (HDL-cholesterol), glucose metabolism (insulin and HOMA-%S) and circulation (systolic blood pressure), but not inflammation (C-Reactive Protein).

A recent systematic review examining the health benefits of physical activity among children and adolescents was unable to draw conclusions on the impact of light-intensity physical activity on health [8]. This was due to the predominant focus on physical activity of at least moderate-intensity in the eligible observational and experimental studies reviewed [8]. Studies to date that have examined the relationship between accelerometer-derived light-intensity physical activity with health outcomes among adolescents [7], [12], [18]–[22], have not observed favourable associations [7], [12], [19]–[21]. For example, while vigorous-intensity physical activity, MVPA, and total physical activity (including movement at all intensities) was associated with markers of total body fat among 365 Spanish adolescents, light-intensity physical activity was not [20]. Similarly, favourable associations were not observed for light-intensity physical activity with body mass index, waist circumference, systolic blood pressure or cardio-respiratory fitness among 605 Canadian youth aged 9 to 17 years [12].

The contrasting findings between previous research and the current study may relate to differences in the thresholds used to define light-intensity physical activity and the tendency to group all light-intensity activities from static (i.e., standing) to dynamic (i.e., slow walking) into a single light-intensity activity variable [7], [12], [18]–[22]. In the present study, when light-intensity was split to represent static (LLPA) and dynamic (HLPA) activities, LLPA and HLPA had some benefit with selected biomarkers; however, after adjusting for MVPA only HLPA had beneficial associations with diastolic blood pressure. Consistent with previous research in adolescents [8], [31], we observed many beneficial associations between MVPA and cardiometabolic biomarkers. Furthermore, the associations for MVPA were stronger when the threshold was placed at 4 versus 3 METs. Collectively, this further supports the evidence that the cardiometabolic health benefits of physical activity tend to increase with the intensity of activity [8]–[12].

While cardiometabolic health benefits tend to increase with the intensity of activity, the volume of physical activity diminishes with increasing intensity. More specifically, the medians in the present study were 250, 102 and 19 min/day respectively for LLPA, HLPA, and MVPA. The 19 min/day median is well below the 60 min/day of MVPA that is recommended for health benefits in the current adolescent physical activity guidelines [1]. Therefore, the findings from this study have potentially important public health implications. While participating in 60 minutes or more of MVPA per day remains the most desirable goal, this may not be realistic for some adolescents. Being more common than MVPA, light activities may be more readily attainable and easier to promote than MVPA, especially in adolescents who are not meeting physical activity recommendations. Furthermore, increasing time in LLPA (e.g., standing) and HLPA (e.g., light ambulatory activities) may be an important first step for increasing MVPA participation in inactive adolescents.

This is the first study to differentiate between the lower and higher ends of the light-intensity activity spectrum when examining associations with biomarkers among adolescents. Further research, including more dose-response studies as well as experimental studies, is needed to confirm and build upon these findings. Such evidence could underpin recommendations regarding light-intensity activity in future physical activity guidelines and inform interventions targeting light-intensity activity, particularly among adolescents for whom MVPA may present challenges.

Strengths of this study include the large sample of adolescents, the objective measurement of physical activity variables, and the examination of several cardiometabolic biomarkers. A major limitation is the cross-sectional design, precluding causal inferences. In addition, while we adjusted for several confounders, residual confounding may have occurred due to unmeasured variables (e.g., pubertal status was not included in the NHANES dataset) or measurement error (e.g., smoking status, dietary recall). Further, a large proportion of participants were excluded due to incomplete data; however, the re-weighting of the data for non-response appears to have minimised any selection bias. Finally, while accelerometers have many advantages over other measures of physical activity, they nevertheless have limitations. For instance, accelerometers do not capture all activities, such as swimming, cycling or load-bearing activities. Additionally, accelerometers require several decisions regarding data reduction procedures (e.g., non-wear time threshold, cut-points) and there is currently a lack of consensus in the literature on the most optimal procedures to use [33]. Furthermore, cut-points, although useful for summarizing the data, underutilise the wealth of information that is captured by accelerometers. New analytic techniques that can identify patterns of behaviour may further extend our understanding of the benefits of physical activity across the intensity spectrum [34].

### Conclusion

Beneficial associations were observed for LLPA and HLPA, in particular with diastolic blood pressure and HDL-cholesterol. Consistent with current physical activity recommendations, MVPA had many favorable associations with cardiometabolic biomarkers among adolescents. The findings from this study suggest light-intensity activities may provide a beneficial adjunct to the current 60 min/day of MVPA recommendation.

## Supporting Information

Table S1 (DOCX)

Table S2 (DOCX)
