# Supplementary material for: Light-Intensity Physical Activity and Cardiometabolic Biomarkers in US Adolescents
Source: PLoS One. 2013 Aug 9;8(8):e71417. doi: 10.1371/journal.pone.0071417 (PMC3739773; doi:10.1371/journal.pone.0071417)
Supplement: Table S1 — (DOCX) [file pone.0071417.s001.docx]

**Table S1.** Associations of time spent in LLPA, HLPA and MVPA (defined as ≥3 METS) with cardiometabolic biomarkers in the full sample (n = 1731)

|  |  | **HLPA (hour/day)** |  | **MVPA (hour/day)** |
| --- | --- | --- | --- | --- |
|  |  | β (95% CI) |  | β (95% CI) |
| Waist Circumference (cm)† |  | 0.002 (-0.002, 0.006) |  | **-0.004 (-0.009, -0.000004)*** |
| Systolic blood pressure (mmHG) |  | -0.104 (-0.467, 0.259) |  | **-0.405 (-0.584, -0.226)*** |
| Diastolic Blood Pressure (mmHG) |  | **-0.464 (-0.840, -0.089)*** |  | -0.275 (-0.522, -0.029) |
| HDL-cholesterol (mmol/L) |  | **0.009 (0.001, 0.016)*** |  | 0.004 (-0.006, 0.014) |
| C-reactive protein (md/dL) † |  | 0.005 (-0.024, 0.034) |  | 0.014 (-0.018, 0.046) |

HLPA = high light-intensity physical activity; MVPA = moderate- to vigorous-intensity physical activity; and HDL = High-density lipoprotein cholesterol.

*** *P* <0.05**; †Log transformed; β (95% CI) = unstandardized regression coefficients and 95% confidence intervals

All Models are adjusted for age, sex, ethnicity, SES, smoking, total energy intake, sodium, saturated fat, and waist circumference (except when the outcome).
