# Supplementary material for: Light-Intensity Physical Activity and Cardiometabolic Biomarkers in US Adolescents
Source: PLoS One. 2013 Aug 9;8(8):e71417. doi: 10.1371/journal.pone.0071417 (PMC3739773; doi:10.1371/journal.pone.0071417)
Supplement: Table S2 — (DOCX) [file pone.0071417.s002.docx]

**Table S2.** Associations of time spent in LLPA, HLPA and MVPA (defined as ≥3 METS) with cardiometabolic biomarkers in the fasting sub-sample (n = 807)

|  |  | **HLPA (hour/day)** |  | **MVPA(hour/day)** |
| --- | --- | --- | --- | --- |
|  |  | Β (95% CI) |  | Β (95% CI) |
| Triglycerides (mmo1/L)† |  | 0.001 (-0.005, 0.004) |  | 0.001 (-0.007, 0.008) |
| LDL-cholesterol (mmol/L)† |  | 0.00001 (-0.006, 0.006) |  | 0.001 (-0.011, 0.001) |
| Plasma Glucose (mmol/L) |  | **0.005 (0.001, 0.010)*** |  | -0.001 (-0.004, 0.003) |
| Insulin (pmol/L)† |  | 0.008 (-0.016, 0.031) |  | -0.015 (-0.035, 0.006) |
| HOMA-%B (n = 768)† |  | -0.005 (-0.023, 0.014) |  | -0.013 (-0.027, 0.001) |
| HOMA-%S (n=768)† |  | -0.010 (-0.030, 0.010) |  | 0.011 (-0.001, 0.024) |
| **OGTT sub-sample (n= 359)** |  |  |  |  |
| 2h plasma glucose, mmol/l |  | -0.009 (-0.078, 0.059) |  | -0.042 (-0.118, 0.034) |

HLPA = high light-intensity physical activity; MVPA = moderate- to vigorous-intensity physical activity; LDL = Low-density lipoprotein cholesterol; HOMA-%B = homeostatic model assessments of β–cell function; HOMA-%S homeostatic model assessments of insulin sensitivity; and OGTT = Oral glucose tolerance test.

*** *P* <0.05;** †Log-transformed; β (95% CI) = unstandardized beta coefficients and 95% confidence

All models are adjusted for age, sex, ethnicity, smoking, total energy intake, sodium, saturated fat, and waist circumference
